# Swimming Exercise Prevents Fibrogenesis in Chronic Kidney Disease by Inhibiting the Myofibroblast Transdifferentiation

**DOI:** 10.1371/journal.pone.0037388

**Published:** 2012-06-27

**Authors:** Chiung-Chi Peng, Kuan-Chou Chen, Chiu-Lan Hsieh, Robert Y. Peng

**Affiliations:** 1 Graduate Institute of Clinical Medicine, College of Medicine, Taipei Medical University, Taipei, Taiwan; 2 Department of Physical Therapy, Graduate Institute of Rehabilitation Science, China Medical University, Taichung, Taiwan; 3 Graduate Institute of Rehabilitation Science, China Medical University, Taichung, Taiwan; 4 Department of Urology, School of Medicine, College of Medicine, Taipei Medical University, Taipei, Taiwan; 5 Department of Urology, Shuang Ho Hospital, Taipei Medical University, Zhonghe, Taipei, Taiwan; 6 Graduate Institute of Biotechnology, National Changhua University of Education, 1, Changhua City, Taiwan; 7 Research Institute of Biotechnology, Hungkuang University, Shalu County, Taichung, Taiwan; Pennington Biomedical Research Center, United States of America

## Abstract

**Background:**

The renal function of chronic kidney disease (CKD) patients may be improved by a number of rehabilitative mechanisms. Swimming exercise training was supposed to be beneficial to its recovery.

**Methodology/Principal Findings:**

Doxorubicin-induced CKD (DRCKD) rat model was performed. Swimming training was programmed three days per week, 30 or 60 min per day for a total period of 11 weeks. Serum biochemical and pathological parameters were examined. In DRCKD, hyperlipidemia was observed. Active mesangial cell activation was evidenced by overexpression of PDGFR, P-PDGFR, MMP-2, MMP-9, α-SMA, and CD34 with a huge amount collagen deposition. Apparent myofibroblast transdifferentiation implicating fibrogenesis in the glomerular mesangium, glomerulonephritis and glomeruloscelorosis was observed with highly elevated proteinuria and urinary BUN excretion. The 60-min swimming exercise but not the 30 min equivalent rescued most of the symptoms. To quantify the effectiveness of exercise training, a physical parameter, i.e. “the strenuosity coefficient” or “the myokine releasing coefficient”, was estimated to be 7.154×10^−3^ pg/mL-J.

**Conclusions:**

The 60-min swimming exercise may ameliorate DRCKD by inhibiting the transdifferentiation of myofibroblasts in the glomerular mesangium. Moreover, rehabilitative exercise training to rescue CKD is a personalized remedy. Benefits depend on the duration and strength of exercise, and more importantly, on the individual physiological condition.

## Introduction

Chronic kidney disease (CKD) usually is associated with impaired cardiac and vascular functions, reduced muscle mass, attenuated muscle strength and power, and an apparent decreased tolerance to exercise [1]. Increasing awareness has prescribed exercise designed to restore certain extent of physical performance and quality of life under those conditions. Numerous interventions involving the aerobic and the resistance exercise training, either the treadmill running or swimming, have been reported beneficial to CKD [2], [3], [4]. Appreciating and understanding the function of exercise is vital to understanding how to prevent CKD which leads to end stage renal disease (ESRD). Pedersen & Fischer indicated strenuous exercise stimulates the myokine (interleukin-6, IL-6) release. The myokine IL-6 in priority is consumed intramuscularly under the influence of AMPK [5]. Different strength and duration of exercise can differentially release different kinds of cytokines [5], [6], [7]. Highly trained athletes usually exhibit a chronic mild hypercortisolism and activation of proinflammatory cytokine IL-6 [8]. IL-6 and TNF-α are produced as a consequence of inflammation [9]. Under many pathological conditions, IL-6 and TNF-α usually are counteracting [10], [11], [12].

PDGF is one of important cytokines involved in mesangial proliferation and renal fibrogenesis in glomerulonephritis and anti-Thy1 nephritis [13], [14]. Almost all experimental and human renal diseases are characterized by altered expression of components of the PDGF system. Infusion or systemic overexpression of PDGF-B or PDGF-D induces prominent proliferative changes of mesangial and renal fibrosis [4]. While the action of PDGF is determined by the relative expression of PDGF alpha-receptors (PDGFR-α) and beta-receptors (PDGFR-β) on the surface of myofibroblasts. These receptors are induced during fibrogenesis, thereby amplifying biological responses to PDGF [7].

PDGFR and alpha-smooth muscle actin (α-SMA) are two markers of mesangial cell activation. Both signs are significantly correlated with the interstitial damage (interstitial infiltrate and fibrosis) [15].

In normal kidneys and in patients with mild histological lesions, the interstitial area showed scattered peritubular cells positive for PDGFR-β and α-SMA, with distribution resembling the capillary network. α-SMA is strikingly increased in patients with moderate to severe lesions, particularly in areas of tubulointerstitial fibrosis [15].

CD34, a sialomucin-type glycophosphoprotein acting as an adhesion molecule, is a marker of haematopoietic stem cells (HSCs) and leukemic cells [16], [17], [18], [19]. CD34 is concentrated mostly in mesangial area and endothelial surfaces in glomeruli [18]. Overexpression of CD34 reflects the pathogenesis of glomerular alterations (e.g. glomerulonephritis) related to age, diabetes, and the severity of the disease [18], [20], [21]. Matrix metalloproteinase-2 (MMP-2) and -9 (MMP-9) may regulate collagen accumulation in CKD inflammatory sites, thus allowing cyst enlargement and limiting the severity of interstitial fibrosis [22].

It has raised evidences showing that chronic training from middle age to old age increases blood oxidative damage [23]. Exercise tends to increase oxidative stress as evidenced by stimulated production of MDA and concomitant downregulation of superoxide dismutase (SOD) [24], similar effect was reported by Coelho et al. (2010) [25]. Long-term exercise could increase damage markers like protein carbonyl content and lipid peroxidation in plasma and erythrocytes [23]. However, the efficiency may depend on the optimal type, frequency, intensity, and duration of the physical activity [26]. Controversial literature revealed chronic exercise appears to be an effective strategy to attenuate the age-related decline in the elderly [23]. Jia et al. (2012) demonstrated that long-term aerobic exercise could remarkably improve hemorheological property and the oxidative stress with hypercholesterolemia [27]. Overall, sport in general applied at moderate loads has predominantly positive effect on the health of humans especially concerning cardiovascular and metabolic diseases [28]. Swimming exercise at 30°C (considered as a cold exposure) evidently revealed beneficial cold specific changes, like increased dehydrogenase [29] and enhanced Ca^2+^-activated myofibril ATPase activities [30]. In addition, exercise increases blood flow and oxygen supply [31]. Exercise training alters the vascular reactivity, enhances endothelium-dependent and -independent renal vasodilation [32]. Literature also indicated that nitric oxide can play an alternative role affecting the blood flow [31].

Considering swimming exercise may improve the flow of blood, oxygen and nutrients to kidneys, we hypothesize swimming exercise may help maintain renal health by excreting toxic metabolites, suppressing renal inflammation, reducing oxidative stress, and inhibiting fibrogenesis to improve CKD. We performed this study using the doxorubicin-Sprague-Dawley rat CKD model.

## Materials and Methods

### Chemicals and Kits

The biochemical tests conducted with the specific enzymatic colorimetric assay kits were all provided by Roche, either located at Switzerland or USA. The reagents IFCC and P-5-P were used for assay of GOT and GPT. Octacalcium phosphate (OCP) and UV were used for the determination of serum calcium and phosphate ions. For other determinations we used BCG for serum albumin, CHOD for cholesterol, lipase-glycerol oxidase for triglycerides; KINETIC for serum BUN, picric acid for creatinine (Jaffe reaction), colorimetric oxidase for uric acid, and Sirius Red for collagen staining. Doxorubicin (DR) was a product of Pfizer (Milano, Italia). Pro-PREP lysis buffer was purchased from the iNtRON Biotechnology (Seongnam, Korea). The kits for other determinations included SOD and TBARS from Cayman (Michigan, USA), the rat IL-6 EIA Kit from PeproTech (NJ, USA), and the rat TNF-α Kit from the R&D Systems Inc. (MN, USA). The sources of the antibodies used in this experiment were: PDGF Receptor β (1∶1000), phosphor-PDGF Receptor β (1∶1000) and β-actin from Cell Signaling (MA, USA); phosphor-PI3K (1∶500) from Santa Cruz (CA, USA); α-Smooth Muscle Actin (1∶1000) from Sigma-Aldrich Co. (MO, USA); CD34 (1∶400) from Leica (Germany). Chemiluminescent HRP Substrate was the product of Millipore (MA, USA). Sodium dodecyl sulfate (SDS) and polyacrylamide gel (PAGE) were products of Sigma Aldrich (MO, USA).

### Animal CKD Model

This experimental protocol was approved by the Institutional Animal Care and Use Committee (IAUCC), China Medical University (Taichung, Taiwan). The Principles of Laboratory Animal Care (NIH publication) were followed. Thirty six 4-week old Sprague-Dawley adult male rats (BioLASCO Taiwan Co., Ltd. Resources) having body weight 220–250 g were used in the study. In the first week, these rats were fed ordinary laboratory chow and acclimated in the animal room conditioned at 23°C±1°C and RH 50–60% with a 12-h/12-h light/dark cycle. The animals had free access to water and pellet chow containing 1.8–2.2% of calcium, 1.1% of phosphorus and 2650 kcal/kg energy. These rats were randomly assigned to six groups: the Normal sedentary (Normal), the doxorubicin induced CKD (DRCKD) sedentary, the 30 min swimming (Swim 30), the DRCKD+30 min swimming (DRCKD+Swim 30), the 60 min swimming (Swim 60); and the DRCKD+60 min swimming (DRCKD+Swim 60). These six groups were separately housed in twelve colony cages, 3 rats in each.

### Swimming Exercise Training Protocol

In the second week, the pre-swimming exercise for acclimation was started in an experimental swimming pool (30°C, water depth: 44 cm; radius 120 cm). A gradual progression protocol was applied beginning with swimming for 5 min to 10 min, and then gradually extended to 20, 30, 40, 50 min per day. CKD was induced by a single s.c. 7.5 mg/kg of doxorubicin after the pre-swimming acclimation [33]. The actual swimming experiment was started on the next day after the doxorubicin injection according to Osato et al. with slight modification [4]. From the third week on, the rats were subjected to 30 min- and 60 min-swimming training exercises respectively, 3 days/week for a total period of 11 weeks. During the whole course, the sedentary rats were remained in the cage under the same environmental condition and inspected daily. At two-week intervals, after having been collected the blood samples, rats were transferred to metabolic cage, one in each, and the urine samples were collected. The samples obtained were subjected to biochemical and immuno- analyses for a duration of 11 weeks. The body weights were assessed by regularly recording every week. After euthanized, the ratio of kidney weight to body weight (KW/BW) was taken. On finishing the experiment, rats were euthanized and the kidneys were excised and subjected to pathological examinations.

### Serum and Urinary Biochemical Parameters

The serum levels of GPT, GOT, cholesterol, triglycerides, BUN, uric acid, creatinine, and albumin were assayed with each specific kit provided by Roche (Switzerland). The serum calcium and phosphate levels were assayed with the specific kits (Roche, USA) by following the manufacturer’s instruction. The superoxide dismutase (SOD) and the thiobarbituric acid reactive substance (TBARs) were assayed with the commercial ELISA kits provided by Cayman Chemical Co. (Michigan, USA). The optical density was taken by the SYSMEX K-1000 Automated Hematology Analyzer (GMI, MN, USA). The blood cell counting for erythrocytes, leukocytes, and platelets was conducted using the SYSMEX K-1000 Automated Hematology Analyzer (GMI, MN, USA).

### Gelatinolytic Zymography

The expression of matrix metalloproteinases MMP-2 and MMP-9 were assayed according to Leber and Balkwill [34]. Briefly, the plasma was centrifuged for 10 minutes at 3000×g. The supernatant serum (10 µL) was loaded onto a 7.5% SDS-PAGE copolymerized with 0.1% gelatin and subjected to electrophoresis under 100 V for 1.5 h. In order to remove SDS, the gel was washed twice, each time with 2.5% TritonX-100 solution for 30 min, and then rinsed with the incubation buffer (0.05 M Tris-HCl buffer, pH 8.0, 5 mM CaCl_2_ plus 5 mM ZnCl_2_). The mixture was incubated at 37°C overnight. The gel was stained with Coomasie Blue at room temperature for 2 h as directed by Leber and Balkwill [34]. Gelatinases in the serum can be detected as unstained gelatin degraded zones on the gel. The amount expressed was quantified with a densitometer (ImagePro Plus 5.0 Media Cybernetics, Bethesda, MD).

### Cytokine Expression

After the swimming training was completed, the rats were subjected to intraperitoneal ketamine and xylasine anesthesia and the blood samples were immediately withdrawn from the abdominal aorta. The levels of IL-6 and TNF-α were measured by the Rat IL-6 EIA KIT provided by PeproTech Inc. (NJ, USA) and the Rat TNF-α KIT by R&D Systems Inc. (MN, USA) according to the manufacturer’s instruction. The minimal detectable limits instructed by the manufacturers for IL-6 and TNF-α are 62 and 5 pg·mL^−1^, respectively.

### Western Blotting

Frozen samples of renal cortex tissues (approximately 100 mg at −80°C) were homogenized with 1 mL of Pro-PREP lysis buffer (pH 7.2) in a homogenizer (T10 basic, The IKA Company, Germany). The homogenate was centrifuged at 12000×g for 20 min at 4°C and the supernatant was collected as tissue sample lysate. The lysate was heated at 100°C for 10 min before loading and separated on precasted 7.5% SDS-PAGE. The proteins were electrotransferred onto the PVDF membrane in transfer buffer for 1 hour. The nonspecific binding to the membrane was blocked with 5% nonfat milk in TBS buffer for 1 hour at room temperature. The membranes were then incubated for 16 hours at 4°C with various primary antibodies. After extensive washing in TBS buffer, the membranes were incubated for 1 hour at room temperature with the secondary antibody in blocking buffer containing 5% nonfat milk. The PVDF membranes were then washed with TBS buffer and the signals were visualized using the Luminescent Image Analyzer LAS-4000 (Fujifilm, Tokyo, Japan). Levels of PDGFR, P-PDGFR, and α-SMA were analyzed by each specific immunoassay according to the manufacturer’s instruction. β-actin was used as reference protein.

### Glomerular Volume

The glomerular volume was calculated according to Weibel (Eq. 1) [35].
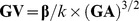
Where GV  =  glomerular volume (mm^3^); GA  =  cross-sectional tuft area (mm^2^); β is the shape coefficient for a sphere (in this paper  = 1.38); ***k*** is the size distribution coefficient (in this paper  = 1.1).

### Histochemical Examination

Kidneys were fixed by immersion with 10% formalin in PBS (pH 7.4) at 4°C for 24 hours and processed for paraffin embedding. Paraffin sections were dewaxed in xylene and rehydrated in a series of ethanol washes. The nuclei of these specimens were stained with Weigert’s Haematoxylin (Sigma-Aldrich, MO, USA), and the collagen content was stained with Sirius Red (Sigma-Aldrich, MO, USA).

### CD34 and α-SMA Immunohistochemical Examination

Paraffin–embedded sections, 3 µm thickness, were deparaffinized in xylene, rehydrated in graded ethanol, and washed in 0.1 mol/L phosphate-buffered saline (PBS, pH = 7.5). The sections were then incubated with 3% H_2_O_2_ for 10 min at room temperature and washed for three times with distilled water. Antigen retrival was performed by heating the sections twice in 200 mL for 1×Antigen Retrival Citrate Buffer in microwave over at 98°C for 8 min. After cooling, the sections were blocked with 1% normal goat serum in PBS for 20 min.

Then the slides were incubated with primary antibodies against α-SMA and CD34 at 4°C for 16 h, and the sections were washed in PBS, incubated with the Post Primary Rabbit anti mouse IgG (Bond™ Polymer Refine Detection kit, Leica Ltd, UK) for 20 min at room temperature. After incubation, the sections were rinsed in PBS and developed by 0.04% 3,3-diaminobenzidine tetrahydrochloride (Bond™ Polymer Refine Detection kit, Leica Ltd, UK). Finally, all sections were then counterstained with hematoxylin and visualized using an Olympus light microscope. Quantitative analysis was performed using an Image–ProPLUS (Meyer Instruments, USA) analysis system at ×400 magnification. Thirty glomeruli in the cortex and the cortex–medulla junctions were randomly scanned. The integrated optical density (IOD) was measured. The sum of the IOD was obtained and the mean value calculated [36].

### Statistics

Data obtained in the same group were analyzed by Duncan’s multiple range tests with computer statistical software SAS 9.0 (SAS Institute Inc., Cary, NC, USA). Significance of difference was judged by a confidence level of *p*<0.05.

## Results

### Body Weight Gain Affected by Swimming Exercise

**Figure 1:**
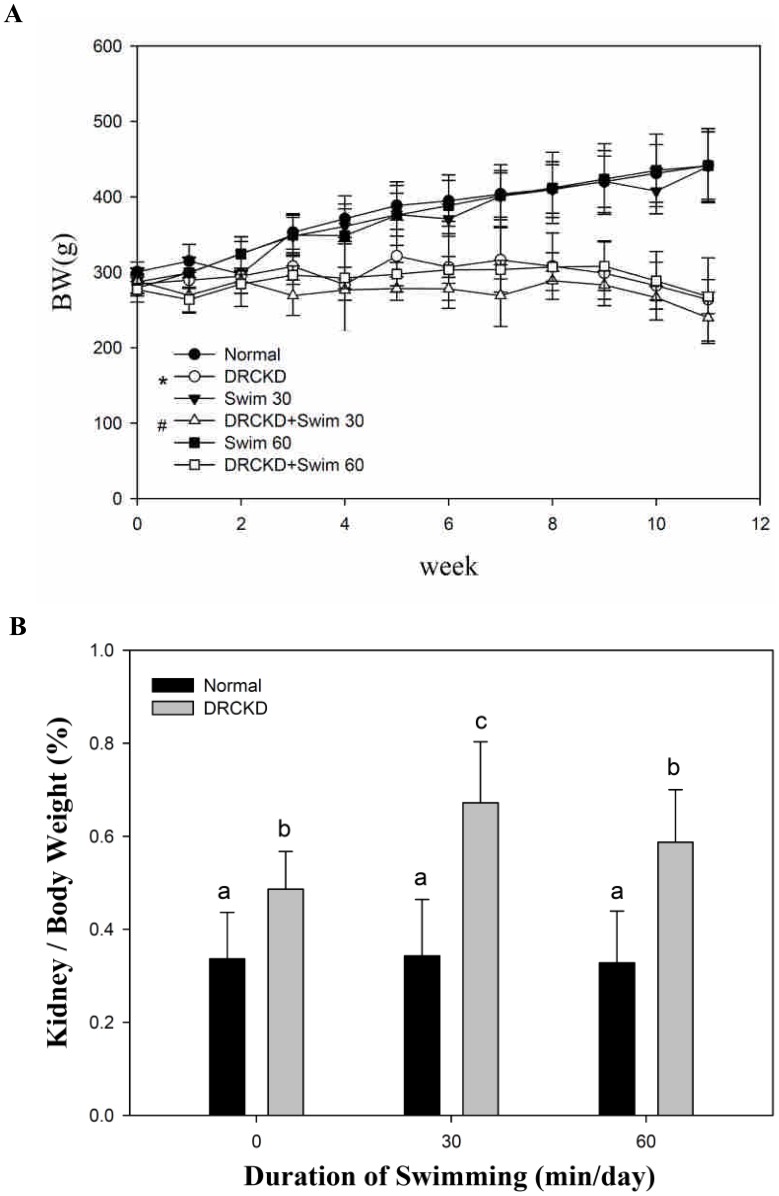
The time course change of body weight (A), the ratio of kidney weight/body weight (B) affected by swimming exercise in different rat groups.

The body weight increase of the three normal controls was very comparable. The body weight increased from 290–300 g at the initial to around 440 g at week 11. All DR-induced rats showed no gain in body weight through the entire course. Conversely, from week 8 on, they showed a slight decline in body weight until to 260 g despite of the exercise or the non-exercise (Fig. 1A).

### The Kidney Weight Affected by Swimming Exercise

All DR-induced rats showed serious swelling of kidneys due to renal edema. The ratio of kidney to body weight (Kw/Bw) for the sedentary group was 0.33. The value was raised to 0.49, 0.67 and 0.59 in groups DRCKD, DRCKD+30 min and DRCKD+60 min swimming exercise, respectively. Apparently, although the 60 min swimming showed better effect, swimming exercise could only partially ameliorate the renal edema status (Fig. 1B).

### Effect of Swimming Exercise on the Appearance of Rat Kidneys

**Figure 2:**
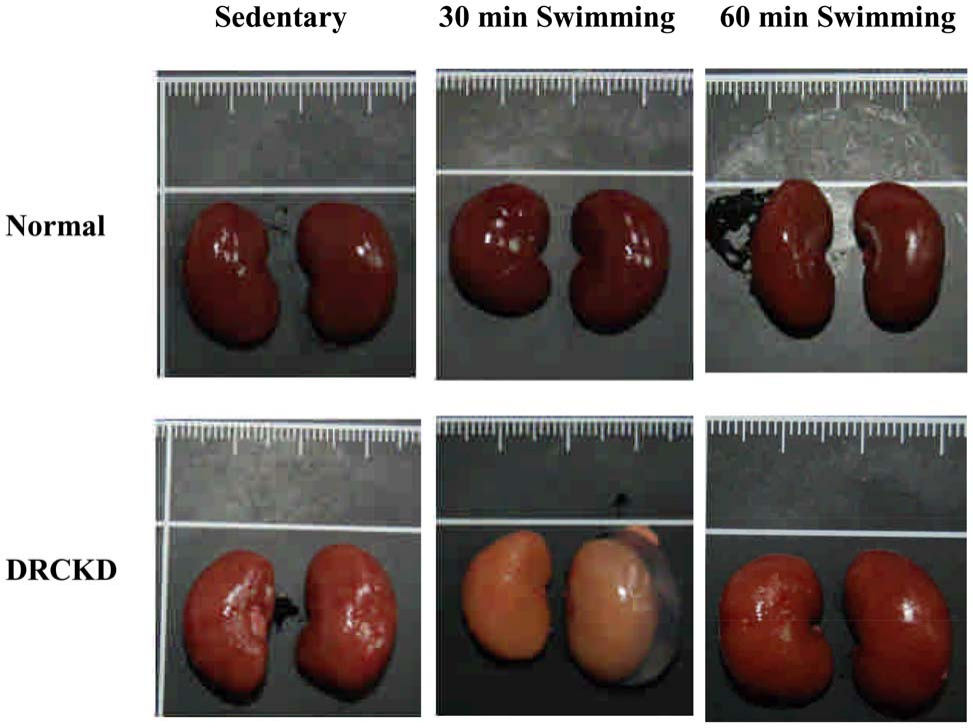
Damages of kidneys induced by doxorubicin secured by swimming exercise.

The outer appearance of normal rat kidneys revealed regular reddish color, contrasting with those pale and whitish kidneys of DRCKD rats (Fig. 2). Serious cortex swelling with large liquid-containing cysts were found in the kidneys of 30 min swimming rats, indicating that 30 min swimming exercise failed to alleviate the DRCKD status (Fig. 2). As contrast, the DRCKD+60 min swimming exercise had improved to some extent, although still remaining swollen with edema in the final stage of experiment, much of the physiological and biochemical parameters shown in the following sections revealed that the 60 min swimming exercise in fact displayed better outcome.

### Swimming Exercise only Partially Restored the Swollen Glomeruli

**Figure 3:**
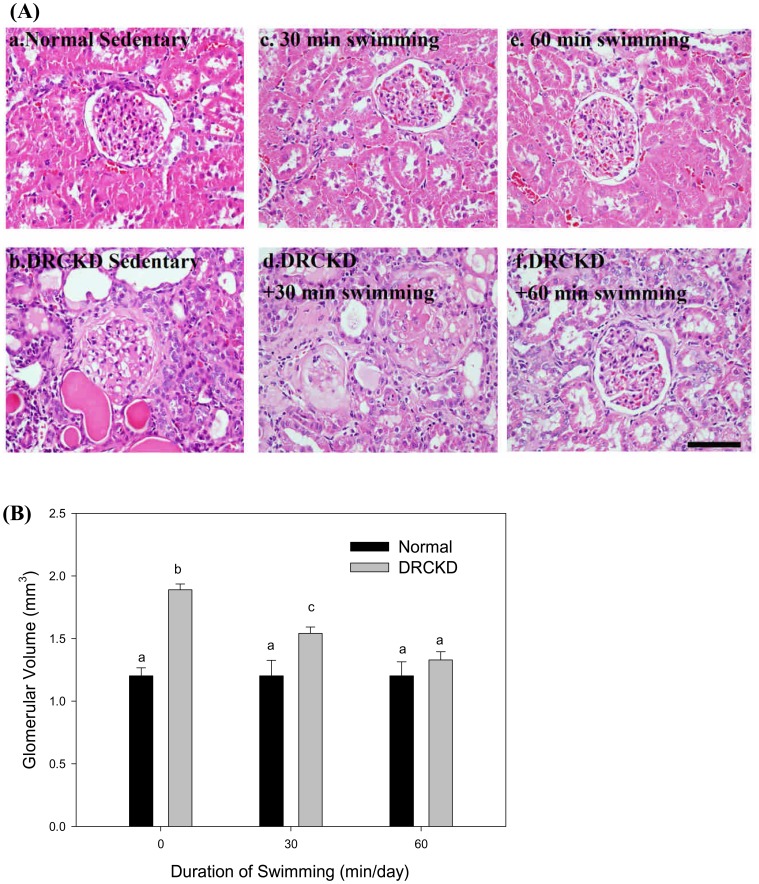
Histochemical examination on the renal cells by Hematoxylin-Eosin staining (A) and the glomerular swelling status (glomerular volume) (B) secured by swimming exercise (magnification ×400). (3A)The six illustrations are: (a) the Normal sedentary; (b) DRCKD sedentary; (c) 30 min swimming; (d) DRCKD+30 min swimming; (e) 60 min swimming; (f) DRCKD+60 min swimming. The pathological findings are: focal, minimal to severe/high, chronic progressive nephrosis (CPN) accompanied with renal cell necrosis, regeneration, hyaline casts, polycysts, and membranous glomerulonephritis with interstitial fibrosis. The glomerular tubules were destroyed by the inflammatory edema and swelling (b and d). The partial recovery by 30 min swimming exercise (d), but more completely secured by the 60 min swimming exercise training (f), in which the DRCKD (b) has been greatly improved to a status with only slight to severe minimal membranous glomerulonephritis and interstitial fibrosis (magnification ×400) (bar = 100 µm).

DR induced renal edema. The glomeruli were swollen to almost destroyed and the surrounding mesangial tissues also became thickened due to swelling. Only the 60 min swimming exercise was shown able to restore most of the status (Fig. 3A). Quantitatively, the glomerular volume of all three normal groups remained unchanged at 1.20 mm^3^, DR significantly increased the glomerular volume to 1.89 mm^3^ with apparent edema and inflammation [5]. On receiving swimming training, the values for the 30 min and 60 min swimming groups reduced to 1.54 and 1.33 mm^3^, respectively, implicating that 60 min swimming exercise was feasibly beneficial to restore the renal edema status (Fig. 3B).

### Effect of Collagen Deposition in Renal Interstitial Tissue by Swimming Exercise

**Figure 4:**
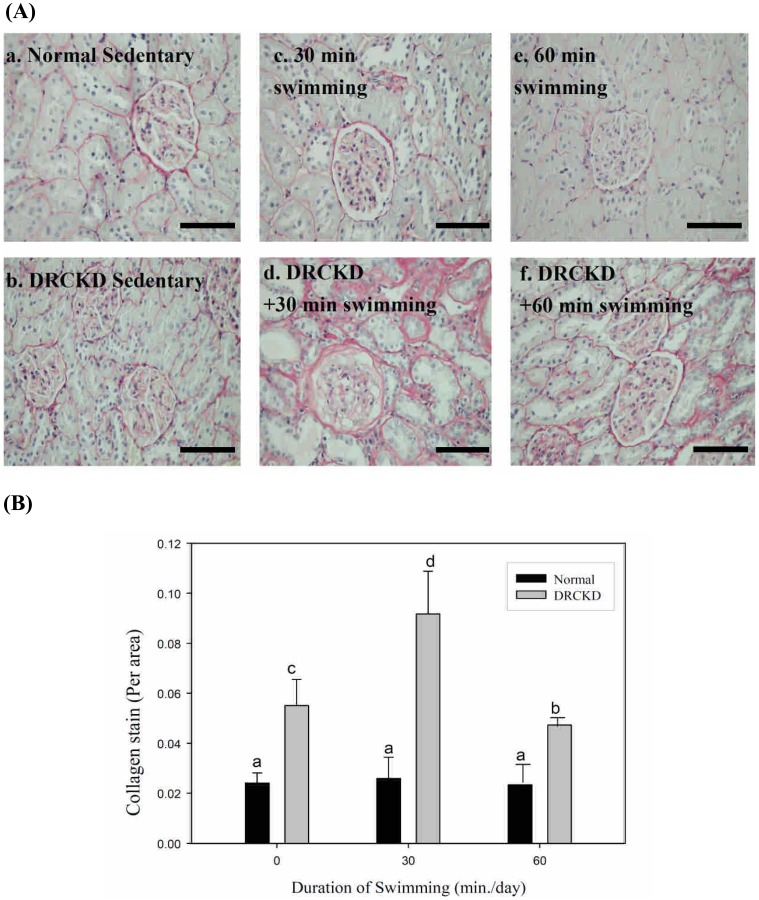
Histochemical examination on the collagen deposition in renal interstitial tissues by Sirius Red stain. (A) Collagen deposition in the renal interstitial tissues (shown in red coloration) was secured by swimming exercise (Magnification ×400). The six illustrations are: (a) the Normal sedentary; (b) DRCKD sedentary; (c) 30 min swimming; (d) DRCKD+30 min swimming; (e) 60 min swimming; (f) DRCKD+60 min swimming. In the early stage of CKD (b), the area of collagen deposition was broader than the normal sedentary group (a). The 30-min swimming exercise enhanced (d), but the 60 min swimming exercise ameliorated such pathological event (f) (magnification ×400) (bar = 100 µm). **(B)** The spectral density of Sirius staining was quantified by Image ProPLUS.

DR induced huge amount of collagen deposition in the interstitial tissue of kidney (Fig. 4A, b) accompanied with very large extent of glomerular swelling, only the 60 min swimming exercise was able to ameliorate the deposition (Fig. 4A, B, C, D, E, F) when compared with Fig. 4A, B, C, D. The spectral density of Sirius staining was quantified as Figure 4B.

### MMP-9 Expression Affected by Swimming Exercise

**Figure 5:**
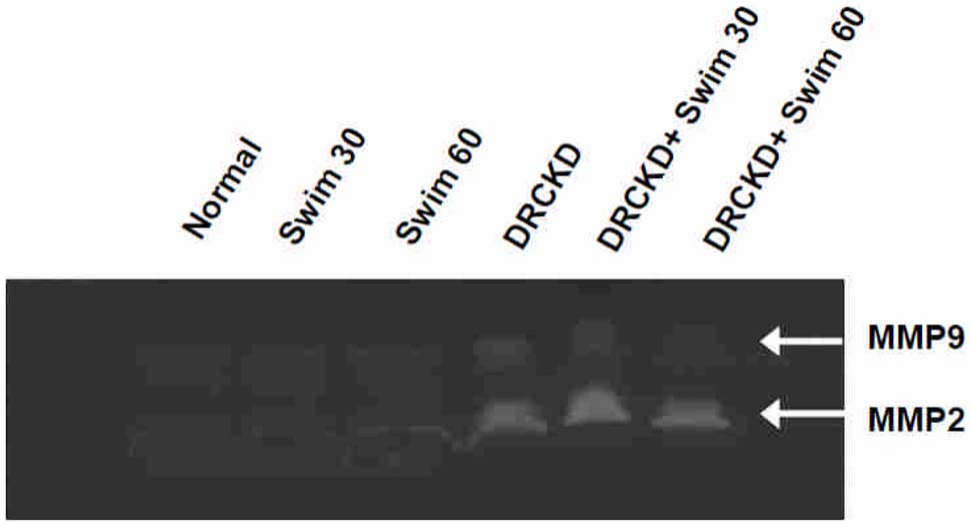
The gelanolytic zymography of the matrix metalloproteinases, MMP-2 and MMP-9. The level of MMP-9 induced by doxorubicin was secured by swimming exercise.

The matrix metalloproteinases MMP-2 and MMP-9 were all shown upregulated by DR, MMP-9 was completely, while MMP-2 was only slightly suppressed by the 60 min swimming exercise (Fig. 5).

### Serum Interleukin-6 and TNF-α Levels Affected by Swimming Exercise

**Figure 6:**
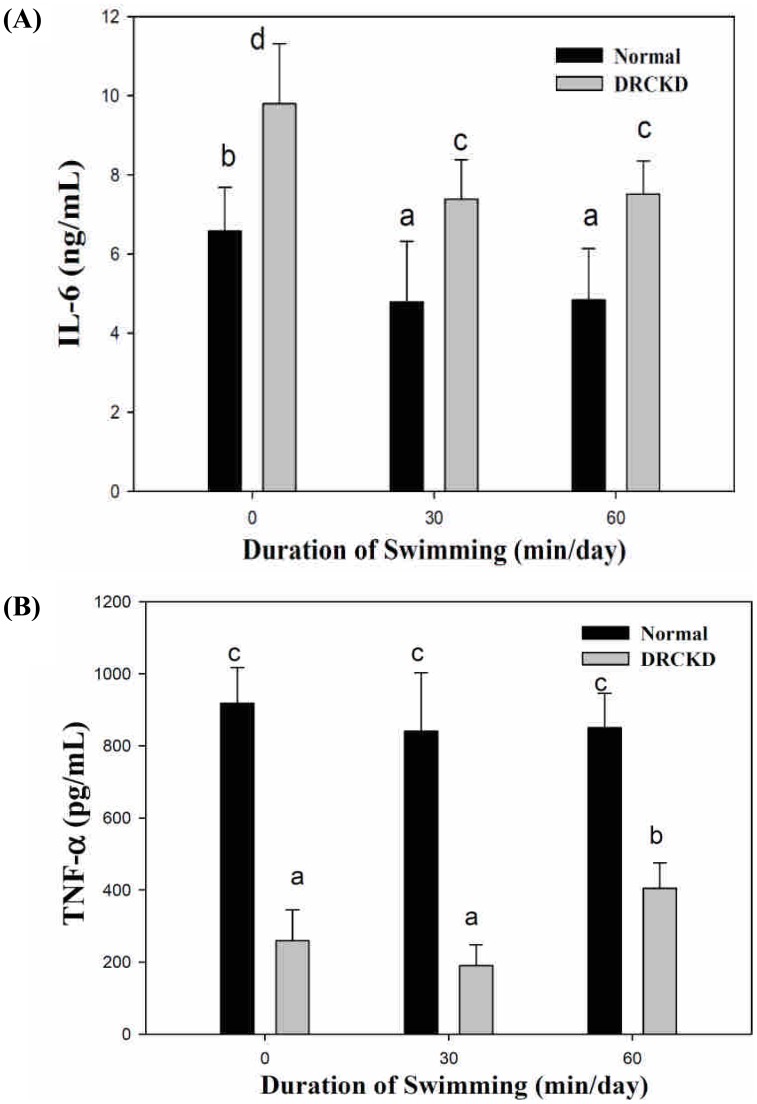
Levels of serum IL-6 (A) and tissue TNF-α (B) affected by swimming exercise.

DRCKD significantly upregulated production of IL-6 to 9.8 ng/mL. Either the 30 min- or the 60 min swimming exercise was shown able to downregulate the level to 7.4–7.5 ng/mL. When compared with the three normal groups, swimming exercise was found unable to restore the level of IL-6 (Fig. 6A). As a contrast, the level of the normal sedentary renal tissue TNF-α was 918 pg/mL and that of DRCKD sedentary was 260 pg/mL. The 60 min swimming exercise was seen able to upregulate its level to 405 pg/mL only, which was still far below the value of the 60 min swimming group, 850 pg/mL. In summary, the 60 min swimming exercise was able to partially improve but could not have completely rescued the DRCKD damages in view of the expression of IL-6 and TNF-α (Fig. 6B).

### Serum GPT and GOT Levels Affected by Swimming Exercise

Although at week 11, DRCKD groups showed slightly higher levels of serum GPT and GOT, reaching respectively 104 U/mL and 176 U/mL, however, by referring to the normal ranges 28–132 U/mL and 59–247 U/mL for GPT and GOT respectively, swimming exercise did not show any effect on the level of these two parameters (Table 1).

**Table 1:** Serum GPT and GOT Affected by Swimming Exercise.* ^,^ †

Parameter	Week 4	Week 11
***Serum GPT (U/mL)***		
Normal	44±5^b,A^	58±4^d,C^
DRCKD	49±5^c,A^	104±7^e,D^
30 min swimming	44±4^b,B^	41±4^a,A^
DRCKD+30 min swimming	44±3^b,A^	54±5^c,B^
60 min swimming	38±4^a,A^	40±5^a,B^
DRCKD+60 min swimming	50±6^c,B^	46±4^b,A^
***Serum GOT (U/mL)***		
Normal	93±4^e,A^	96±5^d,B^
DRCKD	57±5^a,A^	176±9^e,D^
30 min swimming	74±4^c,A^	79±6^c,B^
DRCKD+30 min swimming	62±5^b,C^	39±4^a,B^
60 min swimming	85±6^d,B^	77±7^c,A^
DRCKD+60 min swimming	83±6^d,C^	41±5^b,A^

* Different superscripts in upper case in the same row indicate significant difference between time intervals. Different superscripts in lower case in the same column indicate the significant difference between different groups (n = 6). Significant level *p*<0.05. Data for week 0 were unavailable**.**

† Normal ranges for GOT and GPT are 59–247 U/mL and 28–132 U/mL, respectively. (data source from the National Laboratory Animal Center, Taipei, Taiwan).

### Serum Calcium and Phosphate Levels Affected by Swimming Exercise

Similar result was found for serum calcium. Swimming did not alter the level of serum calcium when compared with the normal range 5.3–13.0 mg/dL (Table 2). In contrast, the serum phosphate level of was slightly elevated in groups DRCKD and DRCKD+30 min swimming, reaching 13.6 and 14.4 mg/dL, respectively. DRCKD+60 min swimming exercise significantly reduced the serum phosphate level to 8.6 mg/mL, almost approaching the normal value 5.3–8.3 mg/dL (Table 2).

**Table 2:** Serum biochemical parameters affected by swimming exercise.*^,†^

Parameter	Week 0	Week 11
***Serum calcium ion (mg/dL)***		
Normal	11.4±1.2^a,C^	11.3±1.2^c,C^
DRCKD	11.3±1.3^a,C^	9.8±0.6^a,A^
30 min swimming	11.4±1.2^a,C^	10.7±1.0^b,B^
DRCKD+30 min swimming	11.2±1.2^a,C^	10.7±1.1^b,B^
60 min swimming	11.2±1.4^a,C^	10.6±1.0^b,B^
DRCKD+60 min swimming	11.4±1.5^a,D^	12.0±1.0^d,C^
***Serum phosphate (mg/dL)***		
Normal	9.9±0.6^b,D^	9.2±0.8^b,C^
DRCKD	10.3±1.4^c,D^	13.6±1.7^d,E^
30 min swimming	9.2±1.2^a,C^	9.4±0.7^c,C^
DRCKD+30 min swimming	9.3±1.3^a,B^	14.4±1.8^e,D^
60 min swimming	10.1±1.5^c,D^	9.1±0.6^b,C^
DRCKD+60 min swimming	9.5±1.4^a,C^	8.6±0.5^a,B^
***Serum cholesterol (mg/dL)***		
Normal	79±2^b,D^	44±2^b,B^
DRCKD	75±2^a,A^	238±14^d,B^
30 min swimming	80±2^b,E^	41±2^a,B^
DRCKD+30 min swimming	82±2^c,A^	350±14^e^
60 min swimming	96±3^e,E^	50±2^c,B^
DRCKD+60 min swimming	87±3^d,A^	348±13^f,D^
***Serum triglyceride (mg/dL)***		
Normal	38±2^c,C^	42±2^b,D^
DRCKD	34±2^b,A^	170±3^d,B^
30 min swimming	33±1^a,A^	44±2^c,C^
DRCKD+30 min swimming	32±2^a,A^	192±7^e,B^
60 min swimming	39±3^d,B^	41±2^a,C^
DRCKD+60 min swimming	45±3^e,A^	255±11^f,C^

### Serum Cholesterol and Triglyceride Levels Affected by Swimming Exercise

The serum cholesterol and triglyceride were all raised by DR induction, reaching 238, 350, and 348 mg/dL, respectively in groups of DRCKD, DCKD+30 min swimming, and DRCKD+60 min swimming. Comparing with the normal sedentary (44 mg/dL), the 30 min swimming (41 mg/dL), and the 60 min swimming group (50 mg/dL), neither swimming exercise was able to ameliorate the hyperlipidemic status induced by DRCKD in comparison with the normal level for cholesterol, 40–130 mg/dL; and 26–145 mg/dL for triglyceride) (Table 2).

### Serum BUN Affected by Swimming Exercise

The DRCKD raised serum BUN level up to 94 mg/dL at week 11. Moderate swimming exercise (30 min swimming) failed to suppress the elevation of BUN. While the aerobic 60 min swimming exercise only partially suppressed the serum level of BUN to 48 mg/mL, 2 folds over the normal range 15–21 mg/dL (Table 3).

**Table 3:** Serum biochemical parameters affected by swimming exercise* ^,^ † (continued).

Parameter	Week 0	Week 11
***Serum BUN (mg/dL)***		
Normal	15±3^d,B,^	14±2^a,A^
DRCKD	13±1^c,A^	94±4^e,E^
30 min swimming	12±1^b,A^	17±2^b,C^
DRCKD+30 min swimming	13±1^c,A^	100±5^f,E^
60 min swimming	13±1^c,A^	18±2^c,C^
DRCKD+60 min swimming	10±1^a,A^	48±3^d,E^
***Serum uric acid (mg/dL)***		
Normal	3.4±0.2^b,B^	2.0±0.1^c,B^
DRCKD	2.7±0.1^a,D^	2.1±0.1^c,B^
30 min swimming	3.3±0.2^b,D^	1.4±0.1^a,C^
DRCKD+30 min swimming	4.8±0.2^e,D^	3.0±0.1^d,C^
60 min swimming	3.8±0.2^c,C^	1.7±0.1^b,B^
DRCKD+60 min swimming	4.6±0.3^d,D^	2.0±0.1^c,A^
***Serum creatinine (mg/dL)***		
Normal	0.5±0.1^a,A^	0.5±0.1^a,A^
DRCKD	0.7±0.1^b,A^	1.0±0.1^c,D^
30 min swimming	0.5±0.1^a,A^	0.5±0.1^a,A^
DRCKD+30 min swimming	0.5±0.1^a,A^	1.1±0.1^d,D^
60 min swimming	0.5±0.1^a,A^	0.8±0.1^b,D^
DRCKD+60 min swimming	0.5±0.1^a,A^	1.1±0.1^d,C^
***Serum albumin (g/dL)***		
Normal	4.4±0.7^c,C^	3.5±0.7^e,A^
DRCKD	4.4±0.5^c,C^	2.4±0.6^a,A^
30 min swimming	4.4±0.6^c,D^	3.4±0.6^d,A^
DRCKD+30 min swimming	4.1±0.7^b,D^	2.2±0.4^a,A^
60 min swimming	4.4±0.7^c,D^	3.4±0.7^d,A^
DRCKD+60 min swimming	4.0±0.4^a,E^	2.6±0.5^c,A^

* Different superscripts in upper case in the same row indicate significant difference between time intervals. Different superscripts in lower case in the same column indicate the significant difference between different groups (n = 6). Significant level *p*<0.05.

† The normal serum levels are: calcium ion, 5.3–13.0 mg/dL; phosphate, 5.3–8.3 mg/dL; cholesterol, 40–130 mg/dL; triglyceride, 26–145 mg/dL; BUN, 15–21 mg/dL; uric acid, 1–6 mg/dL (human standard); creatinine, 0.2–0.8 mg/dL; albumin, 3.8–4.8 g/dL (data source from the National Laboratory Animal Center, Taipei, Taiwan).

### Serum Uric Acid Level Affected by Swimming Exercise

The serum uric acid levels in all groups were not affected by swimming exercise, all remaining within the normal range 1–6 mg/dL (Hospital clinical data) (Table 3).

### Swimming Exercise Failed to Restore the Serum Creatinine Level

DR induced slight elevation of serum creatinine, the levels raised to 1.0–1.1 mg/dL at week 11. Swimming exercise did not show any effect on its restoration when referring to the normal serum creatinine range for SD rats 0.2–0.8 mg/dL (Table 3).

### Swimming Exercise was Unable to Restore Serum Albumin Decrease

DR induced hypoalbuminemia. At week 11, the serum albumin levels were all decreased in the DRCKD rats, ranging from 2.4–2.6 g/dL (Table 3). Swimming exercise failed to restore this trend comparing with the normal range 3.4–4.8 g/dL. The levels remained at 2.4, 2.2, and 2.6 g/dL, respectively in groups DRCKD, DRCKD+30 min swimming, and DRCKD+60 min swimming, comparing with 3.5 g/dL exhibited by the normal sedentary group (Table 3).

### Urinary Parameters Were All Improved

Urinary protein, creatinine, and BUN levels were all partially improved but not completely by swimming exercise training at Week 11. The urinary protein was reduced from 828 to 507, and 258 mg/dL in groups DRCKD, DRCKD+30 min swimming, and DRCKD+60 min swimming, comparing to 20–23 mg/dL of the three normal groups (Table 4). The creatinine levels of all groups except the normal sedentary were very comparable, exhibiting values between 51–72 mg/dL. Interestingly, the normal sedentary group still remained at 128 mg/dL comparing to 125–143 mg/dL of the initial values for all groups (Table 4). As for the level of urinary BUN, after swimming exercise, the value of DRCKD group was reduced from 257 mg/dL for DRCKD to 198 and 140 mg/dL in groups DRCKD+30 min- and DRCKD+60 min swimming exercise, respectively (Table 4).

**Table 4:** Urinary biochemical parameters.*

Parameter	Week 0	Week 11
***Protein, mg/dL***		
Normal	20±12^a,A^	24±12^a,A^
DRCKD	22±13^a,A^	828±26^f,B^
30 min swimming	21±11^a,A^	100±24^b,B^
DRCKD+30 min swimming	22±12^a,A^	507±32^e,B^
60 min swimming	23±10^a,A^	113±25^c,B^
DRCKD+60 min swimming	22±14^a,A^	258±32^d,B^
***Creatinine, mg/dL***		
Normal	125±24^b,A^	128±33^e,A^
DRCKD	138±21^c,B^	58±28^b,A^
30 min swimming	128±22^b,B^	51±34^a,A^
DRCKD+30 min swimming	143±34^d,B^	67±27^c,A^
60 min swimming	121±18^a,B^	67±34^c,A^
DRCKD+60 min swimming	125±27^b,B^	72±23^d,A^
***BUN, mg/dL***		
Normal	55±15^a,A^	60±18^a,A^
DRCKD	60±16^a,A^	257±14^d,B^
30 min swimming	60±13^a,A^	62±17^a,A^
DRCKD+30 min swimming	57±15^a,A^	198±14^c,B^
60 min swimming	56±16^a,A^	58±16^a,A^
DRCKD+60 min swimming	55±17^a,A^	140±14^b,B^

* Data expressed in mean±SD from triplicates (n = 6). The superscripts in lower case indicate significance of difference in the same column, and those in upper case indicate significance in the same row.

### Swimming Exercise Sustained Antioxidative Strength and Reduced Oxidative Stress

DRCKD inhibited the activity of SOD to 38 U/mL at week 4, and 45 U/mL at week 11. Although the 30 min swimming group did not show any effect, the 60 min swimming exercise significantly activated the SOD levels to 82 U/mL in the 60 min controls, comparing to 56–62 U/mL of the normal sedentary group (Table 5). Nonetheless in the DRCKD groups, swimming exercise failed to show any beneficial effect, implicating that although swimming exercise is able to strengthen the *in vivo* antioxidative bioactivity in the healthy control groups, it may fail to restore the antioxidative strength like SOD in the DRCKD rats.

**Table 5:** The oxidative stress in DRCKD and antioxidative power of swimming exercise.

Parameter	Week 4	Week 11
***SOD (U/mL)***		
Normal	56±3^d,A^	62±5^d,B^
DRCKD	38±3^b,A^	45±5^c,B^
30 min swimming	56±7^d,A^	60±2^d,B^
DRCKD+30 min swimming	30±6^a,B^	27±2^a,A^
60 min swimming	82±2^e,A^	82±2^e,A^
DRCKD+60 min swimming	45±5^c,B^	42±1^b,A^
***TBARS (µM)***		
Normal	24±1^b,B^	20±5^a,A^
DRCKD	53±3^c,A^	53±3^d,A^
30 min swimming	23±6^a,A^	23±3^b,A^
DRCKD+30 min swimming	80±6^e,A^	100±7^f,B^
60 min swimming	24±5^b,A^	24±2^c,A^
DRCKD+60 min swimming	59±4^d,A^	58±4^e,A^

* Different superscripts in upper case in the same row indicate significant difference between time intervals. Different superscripts in lower case in the same column indicate the significant difference between different groups (n = 6). Significant level *p*<0.05. Data for week 0 were unavailable**.**

Swimming exercise did not show any effect in the 30 min- and 60 min swimming groups. DRCKD significantly raised the level of TBARs to 53 µM comparing to 20–24 µM of the normal sedentary group. Amazingly, the moderate 30 min swimming exercise further enhanced the level to 80 µM at week 4 and to 100 µM at week 11. Conversely, the 60 min swimming exercise suppressed the formation of TBARs significantly to 58–59 µM, an implication in the beneficial effect of aerobic exercise, the 60 min swimming, on the oxidative damages occurring on the lipoproteins like low density lipoprotein (Table 5).

### Swimming Exercise more Prominently Affected Platelet Counts

By referring to the normal red blood cell (RBC) count (7.0–10.0)×10^6^/µL for the Sprague Dawley rats, doxorubicin moderately suppressed the erythrocyte formation, the lowest count was seen with the DRCKD+30 min swimming group (5.8±0.5)×10^6^/µL (Table 6). Unlike the RBC, the leukocyte (the white blood cell, WBC) count in all DRCKD rats revealed to be with normal count except the 30 min swimming exercise, which was raised to high peak count 25±5×10^3^/µL. As a contrast, the WBC level was restored to normal (17±5)×10^3^/µL by the 60 min swimming exercise (Table 6). The reason why the RBC concentration was not apparently affected, while the WBC count in the DRCKD+30 min swimming exercise was so highly raised is worth investigation. Similarly, the platelet count was significantly raised to (1445±589)×10^3^/µL and (1513±137)×10^3^/µL in groups DRCKD and DRCKD+30 min swimming training, respectively. Only the 60 min swimming program was able to restore the platelet count to (1016±475)×10^3^/µL [The normal range is (500–1300)×10^3^/µL] (Table 6).

**Table 6:** Blood cell count affected by swimming exercise.#

Parameter	Blood cell counts at week 11		
Cell	RBC (×10^6^/µL)	WBC (×10^3^/µL)	Platelet (×10^3^/µL)
	(Normal range: 7.0–10.0)#	(Normal range: 6.0–17.0) #	(Normal range: 500–1300) #
Normal	7.4±0.1^b^	10±4^c^	875±136^a^
DRCKD	6.4±1.7^a^	8±0^c^	1445±589^a,b^
30 min swimming	7.6±0.6^b^	13±2^b,c^	796±112^a^
DRCKD+30 min swimming	5.8±0.5^a^	25±5^a^	1513±137^a^
60 min swimming	6.8±1.4^b^	9±3^c^	643±67^a^
DRCKD+60 min swimming	6.4±0.5^b^	17±5^b^	1016±475^a,b^

# Values in each column with different superscripts of lower case (a to c) indicate significantly different with each other at significant level *p*<0.05.

### Swimming Training Downregulated PDGFR and P-PDGFR in DRCKD Rats

**Figure 7:**
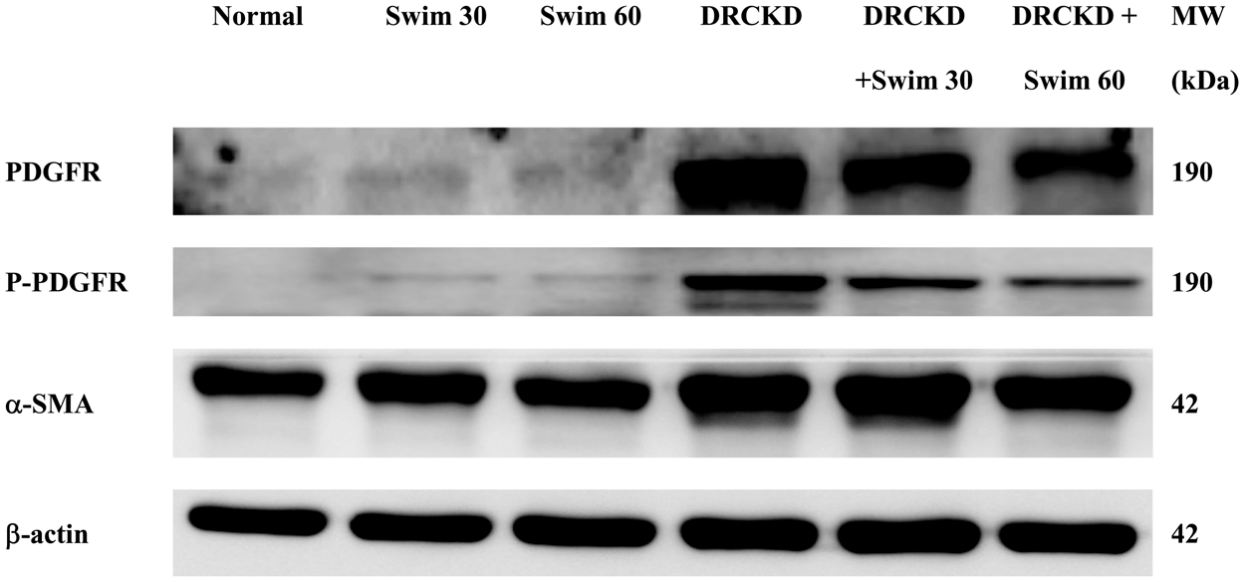
Western blotting for PDGFR, P-PDGFR, and α-SMA in DRCKD rat kidneys which were restored by swimming exercise.

In the three control groups, the levels of PDGF and PDGFR were very comparable. DR apparently upregulated, while swimming exercise downregulated, the levels of PDGFR and P-PDGFR in DR treated groups (Fig. 7).

### Swimming Training Downregulated CD34 and α-SMA in DRCKD Rats

**Figure 8:**
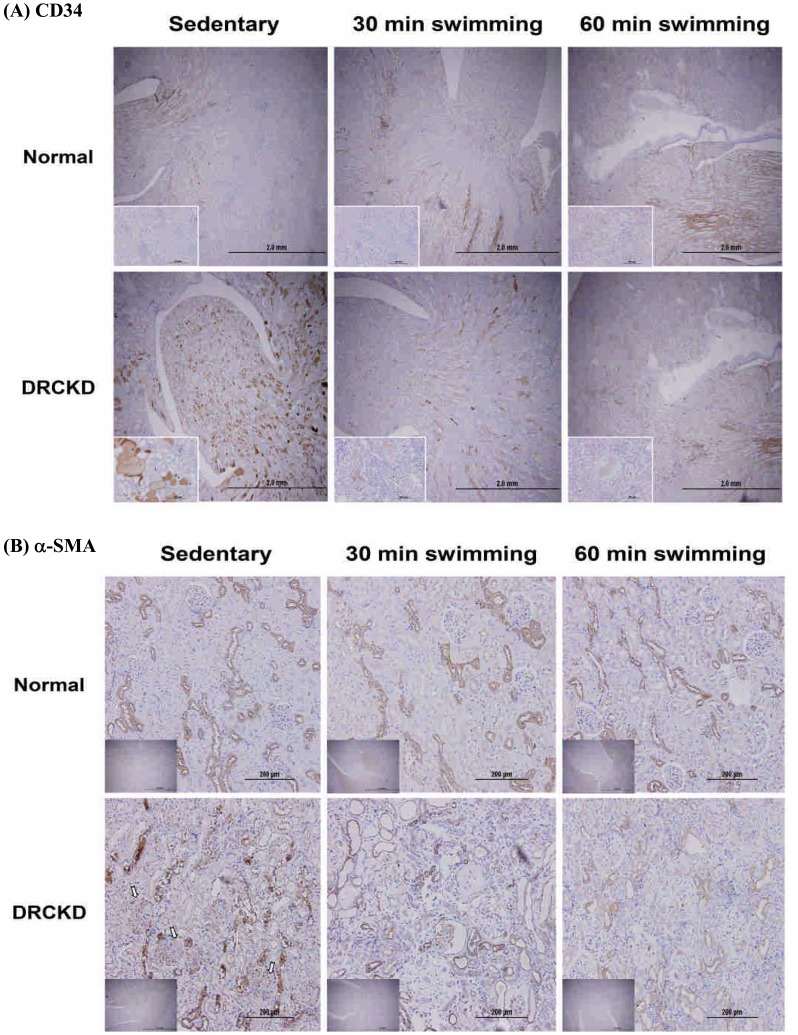
Immunohistochemical examination of CD34 (A) α-SMA (B) indicating myofibroblasts progressing transdifferentiation fibrogenesis. The myofibroblasts are emerging in the mesangium of glomeruli and on the epithelia of the tubule convolutes. (magnification: CD34×40; α-SMA ×200).

Immunohistochemical examination revealed that a tremendous number of myofibroblasts undergoing transdifferential fibrogenesis were apparently emerging mostly in the mesangium of glomerulli, less amount on the epithelia of convoluted tubules, as evidenced by the expression of CD34 (Fig. 8A) and α-SMA (Fig. 7 & 8B). Partial recovery was found in the DRCKD+30 min swimming exercise group, contrasting to the complete amelioration in the DRCKD+60 min swimming exercise subjects (Fig. 8).

## Discussion

### DR Reduced Weight Gain Due to “Protein-energy Malnutrition”

Potential mechanisms of muscle wasting in renal failure can occur through the insulin and IGF-1 receptor-mediated signaling via the insulin receptor substrate (IRS)/phosphoinositide-3 kinase (PI3K)/Akt pathway, which drives anabolic, anticatabolic, and antiapoptotic processes, potentially leading to a catabolic state with body weight loss [2]. Moreover, a reduction in circulating amino acid levels, as is often seen in renal failure patients, would reduce the anabolic stimulus functioning via this pathway as well [2], [9].

**Figure 9:**
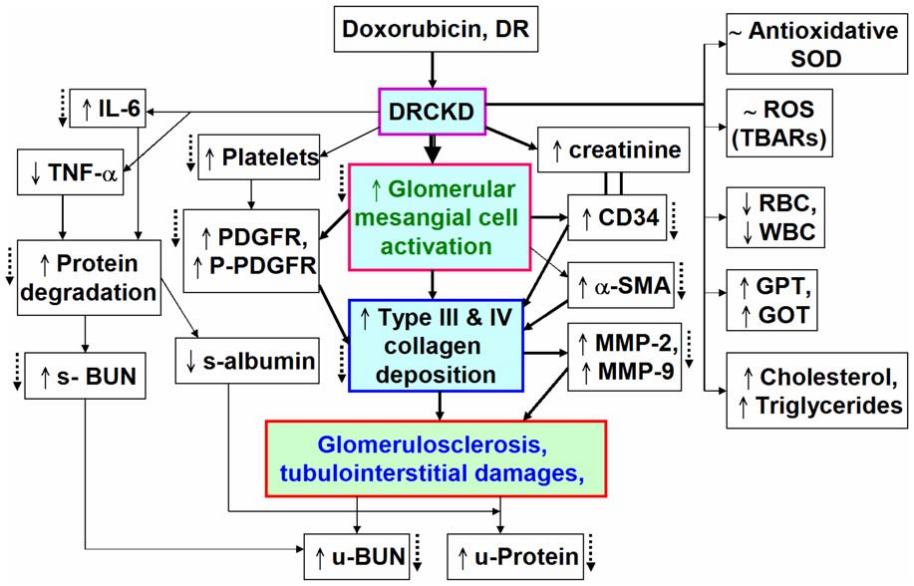
A summary of the signaling in DRCKD and the ameliorating effect of 60-min swimming exercise. DR induced chronic kidney disease (CKD) and activated glomerular mesangial cells, increasing platelets counts, upregulating PDGF and PDGFR, overexpressed CD34, α-SMA and MMP-2 and MMP-9, causing type III and type IV collagen deposition in the interstitium of glomeruli, resulting glomerulosclerosis and tubulointerstitial damages (solid arrows inside the box). 60-min swimming exercise may ameliorate the status of CKD (dotted arrows outside the box).

Low grade exercise or too short the period of exercise could stimulate proinflammatory cytokines known to inhibit directly anabolic activity of GH→IGF-1 axis, resulting in the body weight loss [37]. As seen, 60 min swimming exercise retained higher body weight than the 30 min equivalent (Fig. 1), indicating higher quality of 60 min swimming training in this regard (Fig. 1). As evidenced, severe body weight loss was seen in the DRCKD victims, a status similar to the “protein-energy malnutrition” described by Fock et al. [38].

### Kidneys Enlarged in DRCKD but Glomerular Volume Restored by Swimming Exercise Training

The kidneys of DRCKD rats exhibited severe renal swelling and edema, and more surprisingly, the rats having received swimming exercise revealed much more enlarged kidneys (Fig. 1), implicating the glomerular blood flow had not been recovered by swimming exercise. Speculatively, the blood flow in the kidneys of CKD rats and the GFR improved by swimming exercise elicited a transient swelling in the early stage of recovery [39], [40]. This secondary renal swelling could in turn release or compensate in part the burden caused by DRCKD in the renal interstitial tissue. As evidenced, the ratio Kw/Bw finally was reduced to 0.59 by the strenuous DRCKD+60 min swimming exercise at week 11 (Fig. 2B). Similar trend was seen in the glomerular volume (GV) change (Fig. 3B), underlying the 60 min swimming exercise training exhibiting better effect in improving the DRCKD status.

### Serum and Urinary BUN not Effectively Reduced by Aerobic Exercise Training

As evidenced by serum and urinary BUN, the trend in improving renal function by exercise was apparently observed in DRCKD rats, yet still incomplete (Table 3; Table 4). Exercise positively increases blood flow, oxygen and nutrient transport to kidneys to help maintain renal health [2], [3], an implication in the *in vivo* actively proceeding catabolic nature of DRCKD [2], [9].

### Swimming Exercise Ineffective in Restoring Serum Albumin and Proteinuria

More often, the glomerular filtration rate (GFR) will be greatly reduced in CKD patients, and concomitantly, proteinuria and glomerular hypertension may be evoked [41]. Proteinuria is a risk factor for progression of chronic renal failure (CRF), which is very common in CKD patients [42]. The reasons that swimming exercise failed to ameliorate the serum albumin level may be due to i) the enhanced renal excretion, resulting increased factional clearance of albumin [43], and ii) the increased catabolic effect [2], [9] (Table 3).

### Upregulation of MMP-2 and MMP-9 in DRCKD Reduced by Swimming Exercise

Tubular cell epithelial-mesenchymal transition (EMT) is a fundamental contributor to renal fibrosis. In moderate and severe tubulo-interstitial damage, increased expression of MMP-2 had been noted [44]. MMP-2 may regulate collagen accumulation at those sites (Fig. 4, 5) [45]. During severe tubulo-interstitial damage, highly upregulated MMP-2 may contribute the pathological basement membrane splitting and disruption of type–IV collagen [44]. Similar yet lesser expression was also seen with MMP-9. Thus, swimming exercise was able to partially ameliorate the CKD status.

### Oppositely Regulated Status of IL-6 and TNF-α during Exercise Performing

In some occasion of catabolic status, the chronic inflammation associated with renal failure often can lead to elevated levels of TNF-α and IL-6. But this was not the case with our experiment (Fig. 6). IL-6 is a well known fibroblast growth factor [46], often reported to be associated with fibrosis [47]. Both cytokines are known to induce muscle atrophy. One possible mechanism for this effect is negative modulation of IRS/PI3K/Akt signaling that effectively reduces cellular sensitivity to IGF-1 and insulin [2], [9], [48]. Speculatively, the 60 min swimming exercise could not trigger the production of IL-6 (myokine), hence the total IL-6 level was downregulated by swimming (Fig. 6). Reducing adipose tissue mass, through weight loss in association with exercise, can lower TNF-α and IL-6 levels and increase adiponectin concentrations [49]. Similarly, IL-6 upregulates the number of TNF-alpha receptors, resulting in suppressed hepatic TNF-α levels [50]. Conversely, the mechanism behind the downregulation of adiponectin during exercise had been ascribed to the high quality exercise-induced upregulation of (myokine) IL-6 expression [51], [52], [53]. Swimming significantly downregulated IL-6 or upregulated TNF-α (Fig. 6), an implication in the beneficial effect of swimming exercise. To summarize, different extent of exercise exerts differently in strength [5].

### Swimming Exercise was a Good Hypolipidemic Agent, However Ineffective Toward the Hyperlipidemic Status Induced by DRCKD

In the DRCKD groups, we observed that the cholesterol and triglyceride levels were unsuppressed by swimming exercise, a result contrary to Osato et al. [4]. There is recent evidence of a link between IL-6 and AMP-activated protein kinase (AMPK). AMPK activation stimulates fatty acid oxidation and increases glucose uptake [54]. We speculate that DR may have highly activated the enzymes malonyl transferase and mevalonate kinase, and exercise might have used up some adipose tissue and cholesterol without inhibiting these enzymes (Table 2).

### Doxorubicin Upregulated PDGFR and P-PDGFR, Swimming Exercise Ameliorated Progressive Renal Disease

Growth factors have been demonstrated to be important mediators of extracellular matrix (ECM) accumulation in glomerulonephritis (GN). TGF-β, PDGF and basic fibroblast growth factor have been shown to promote ECM accumulation, tubulointerstitial hyperplasia and fibrosis [55]. Mesangial cells produce PDGF, and various growth factors induce mesangial proliferation via induction of PDGF-B chain synthesis. Glomerular mesangial matrix accumulation characterizes many progressive renal diseases [56], [57]. DR overexpressed PDGFR and P-PDGFR, causing a pre-fibrotic status in kidney (Fig. 3A), while swimming training downregulated PDGFR and P-PDGFR in an intensity-responsive fashion (Fig. 7). Studies have demonstrated that overactivity of PDGF-BB in scarless fetal wounds induces fetal wound fibrosis [22], [58]–[60].

In chronic inflammatory conditions, the stimulatory effect of PDGF on connective tissue cells may lead to tissue fibrosis, consistent with our data (Fig. 3A and 4) [57], to extend, the use of anti-PDGF-B can be a good therapeutic approach to progressive renal disease [56].

### The Antioxidative Nature of Swimming Exercise

Silveira et al. hypothesized that after acute bouts of moderate exercise, activation of the sympathetic nervous system would lead to activation of nuclear factor-κB (NF-κB) biochemical pathways via the Ras/ERK cascade with concomitant rise in NADPH oxidase activity and generation of reactive oxygen species [9], in some circumstances, elevation of cortisol and cathecholamines in plasma could be involved [61].

Superoxide dismutase (SOD) is an *in vivo* cellular antioxidant enzyme which acts as a catalyst in the process of dismutation of superoxide into oxygen and hydrogen peroxide. SOD works as an antioxidant because it outcompetes the damaging reactions of superoxide on genetic material. SOD has been proven to be very effective in the treatment of colonic inflammation. Also, the antioxidant properties of SOD may make it an important treatment for inflammatory bowel disease [62].

DRCKD inhibited the activity of SOD, while DRCKD+60 min swimming group was shown able to partially recover its level. Such a result was totally not seen in the DRCKD+30 min swimming, implicating the aerobic exercise was more effective to evoke the cellular antioxidative capability.

Similarly, DRCKD significantly boosted the level of TBARs to 53 µM comparing to 20–24 µM of the normal sedentary group. The moderate 30 min swimming exercise further enhanced the level to 80 µM at week 4 and to 100 µM at week 11. Conversely, 60 min swimming exercise significantly suppressed the formation of TBARs to 58–59 µM, again implicating the beneficial effect of aerobic exercise (Table 5). Data consistent with Aslan et al. (1998), the MDA level after the 5 week training program was lower than the MDA level after acute exercise period but was still higher than sedentary period [63].

### DRCKD Upregulated CD34 and α-SMA

CD34 is a marker of haematopoietic progenitor cells, stromal precursors, vascular endothelial cells, and a variety of stromal tumour cells. At the level of extraglomerular or intraglomerular mesangium, CD34 may signal either the presence of HSCs, or conversely, may be a marker of transdifferentiation [19]. Immunohistochemical co-staining of CD34 and α-SMA has been used to study various glomerulonephritis (GN) as a transformed mesangial cell marker [64]. In normal glomeruli, all cell types were negative for CD34, but in glomeruli in mesangial proliferative glomerulonephritis, CD34 is expressed exclusively in mesangial cells in parallel to α-SMA expression (Fig. 8A and 8B) [64]. Alternatively, Gluhovschi et al. indicated CD34 does not significantly correlate with mesangial α-SMA [19]. Swimming exercise at 60 min program restored its level to normal (Fig. 8A). Recent studies of Galeano et al. (2007) found that a genetic defect affecting sialic acid biosynthesis causes hematuria, proteinuria, and structural glomerular defects leading to animal death within days after birth [65]. α-SMA is a specific marker of mesangial cell activation [66], while it seems to have a better correlation with serum creatinine [19]. α-SMA plays key roles in glomerular remodeling [67], 60 min swimming training restored it to normal level (Fig. 7, 8B). The experimental results are summarized in Fig. 9.

To quantify the efficiency of different exercises on (myokine) IL-6 release, we elucidated a mathematical model (See ‘Support Information S1”). Mathematical evaluation pointed out that 60 min swimming exercise does not release myokine. Instead, it directly consumes plasma IL-6 in proportional to the work expenditure. The consumption coefficient for swimming exercise **K’**_swim_ was −0.1098 pg/mL-J. While from the 60 min treadmill exercise we showed the “strenuosity coefficient” or “the myokine releasing coefficient” to have a magnitude 7.154×10^−3^ pg/mL-J (See ‘Support Information S1”), which apparently was not achievable by the swimming exercise, implicating the differential benefits being dependent on duration- and strength of exercise.

In summary, exercise represents a physical stress that challenges homeostasis. The challenges including the clinical, the patient’s, systemic and the environment factors involve most of the relevant physiological, biochemical, and immunological parameters. Exercise rehabilitation for CKD patients is a personalized rehabilitation in nature. Bergamaschi et al. (1997) pointed out the participation of other associated factors in the experimental model must be carefully inspected [41], [6]. Thus, an optimum benefit only can be achieved provided a perfect rehabilitation design is available.

### Conclusion

The 60 min swimming exercise training is more effective than the 30 min alternative in improving the DRCKD status. The 60 min swimming exercise prevents fibrosis of glomerular mesangium by inhibiting mesangial cell activation and CD34 expression, and simultaneously, by downregulating IL-6, PDGF, PDGFR, P-PDGFR, α-SMA, and dysregulating MMPs to suppress myofibroblast transdifferentiation. To quantify the critical effective condition for myokine IL-6 release, we have elucidated a mathematical index “The strenuosity coefficient” or “The myokine releasing coefficient” with a magnitude 7.154×10^−3^ pg/mL-J, and only above this value, the myokine IL-6 can be released during exercise.

## Supporting Information

Support Information S1 **Modeling of myokine release resulting from exercise trainings.**
(DOC)
