# Supplementary material for: Swimming Exercise Prevents Fibrogenesis in Chronic Kidney Disease by Inhibiting the Myofibroblast Transdifferentiation
Source: PLoS One. 2012 Jun 27;7(6):e37388. doi: 10.1371/journal.pone.0037388 (PMC3384651; doi:10.1371/journal.pone.0037388)
Supplement: Support Information S1 — Modeling of myokine release resulting from exercise trainings. (DOC) [file pone.0037388.s001.doc]

**Support Information file**

**S1. Modeling of myokine release resulting from exercise trainings**

**S1.1. Quantification of the work strength done by exercises**

Previously, we had performed the treadmill exercise study [23]. In order to understand the myokine release by aerobic strenuous exercise, the following mathematical deduction was used to compare the work equivalent of myokine release by different exercises.

**S1.1.1. *Work done by the treadmill exercise***

Let the body weight of per rat be W_o_ kg and the trainees moved horizontally at a constant speed V m/h along the treadmill whose friction coefficient is 0.30 [68]. When the rat moves along a distance S m at a constant speed V m/h in time of Δt h, we have


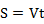
……………………………………………………………………..……...1

As a constant speed means no acceleration, the net force must be zero, and the force applied must equal the force of friction. On a horizontal surface: the normal force F_n_ = weight of subject = mass × gravity. Thus we have


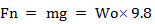
 N …………………………………………..….………2

And the friction force


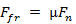
………………………………………………………………………3

Where μ is the friction coefficient, F_n_ is the force normal to the surface of direction the trainees are moving. Substitution of the given parameters into Eq. 3 leads to


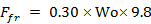
 N ………………………………………….…..……..4

The work done by the trainees is


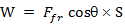
…………………………………………………….………5

Without slope raise, cosθ =1.0, hence the work W_tread_ done in the treadmill exercise would be


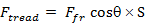


=
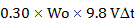
 J ………………………………....................6

Or


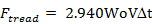
 J …………………………………............................7

**S1.1.2. *Work done by the swimming exercise***

Assuming that 10% of the body (mainly the head) has to be kept over the water surface during swimming, and due to the buoyant force the effective weight W_eff_ would be less in form of


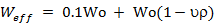
……………………………………..…………8

Or


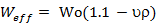
………………………………. ……………………..….9

Where W_eff_ is the effective body weight (kg), W_o_ is the apparent body weight in the air (kg), υ is the specific volume of rat body (in m^3^/kg), ρ is the density of swimming medium (kg/m^3^), which is 1000 kg/m^3^ for water. Taking 0.33 as the friction coefficient for water and assuming the specific volume of rats to be 0.0008 m^3^/kg, substitution of 1000 kg/m^3^ for water into Eq. 9 to give [67]


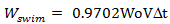
 J……………………………………………….…10

Eq. 10 indicates that the work exerted by the swimming exercise only is one third of that of the treadmill exercise, provided the exercise speed V and duration Δt are kept the same.

Moreover, in response to muscle contractions, both type I and type II muscle fibers express the myokine IL-6, exerting effects in majority locally or intramuscularly through activation of AMPK, thus the muscle strenuosity should be considered to be an another factor crucial to trigger the release of myokine IL-6 [5]. To sum up the mechanisms, we have


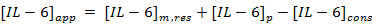
 ..............................11

where [IL-6]_app_ is the apparent IL-6 in the plasma during the exercise training. [IL-6]_m,res_ is the residual myokine IL-6 released into the plasma after a high quality exercise. To approximation, [IL-6]_p_ involves the normal plasma level [IL-6]_n_ and the portion relevantly related to immunological response, mostly inflammation [IL-6]_infl_. [IL-6]_cons_ is the amount of IL-6 consumed in the whole course. Substitution of these new terms into Eq. 11 leads to


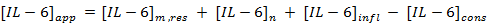
........12

Assume that in a relatively short period the consumption of IL-6 is exclusively by exercise, which in essence is of myokine origin. Thus the total IL-6 released in the muscle during the treadmill exercise (i.e. myokine; [IL-6]_m,t,tread_) would be the sum of these two parts: the consumption term [IL-6]_cons_ and the residual term [IL-6]_m,res_,


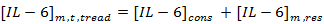
..………………….............…13

Which approximately is proportional to Eq.7 in form of


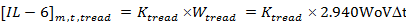
 J……...........14

[IL-6]Similarly for the swimming exercise we have from Eq.10


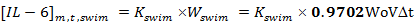
 J …............15

The parameters K_tread_ and K_swim_ are called herein “the myokine releasing coefficient” or more implicitly “the strenuosity coefficient”, which obviously is dependent on the quality of exercise.

Both Eq. 14 and 15 predict that different quality of exercise exerts differently in effectiveness. Recently, the expression of cytokines (e.g. IL-6 and IL-17) and other proinflammatory cytokines were reported to be differentially correlated with the duration, the strength, and the strenuosity of exercise [5, 7, 9, 37]. With which this present mathematical postulation is rather closely consistent.

Theoretically, the normal level [IL-6]_n_ can be obtained from the sedentary group, the inflammation term [IL-6]_infl_ can be derived by substracting the level of [IL-6]_n_ from the IL-6 level of the DRCKD control group. The residual myokine term [IL-6]_m,res_ can be obtained separately from the difference of the exercise control and the sedentary values.

The data for the treadmill exercise were [24]: the IL-6 of the normal sedentary [IL-6]_n_ = 14.9 ng/mL; IL-6 for DRCKD sedentary = 18.7 pg/mL; 30 min exercise control = 21 pg/mL; DRCKD+ 30 min exercise =23 pg/mL. For the 60 min exercise the corresponding values are 16.5 and 17.5 pg/mL. Thus the residual myokine for the 30 and 60 min exercise are 6.1 pg/mL (21-14.9 pg/mL) and 1.6 pg/mL (16.5-14.9 pg/mL). Obviously, the 60 min exercise control utilized the extra myokine 4.5 pg/mL (= 21-16.5 pg/mL) or 5.5 pg/ml (23-17.5 pg/mL) by the DRCKD + 60 min exercise than the 30 min exercise group [24]. From these data, the consumption rate per min exercise per rat weighing 280 g was


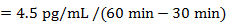


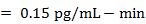
.........................................................................................16

For 60 min exercise the total consumption of myokine IL-6 per rat was


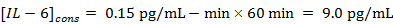
............................17

and the residual myokine was


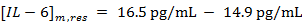


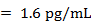
................................................................................18

Substitution of these data into Eq. 13 gives


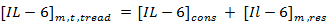


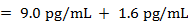


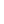

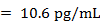
.....................................................................................................19

Given in the speed of treadmill V = 30 m/min, substitution of this value and the total myokine released during the 60 min treadmill exercise 10.6 pg/mL (Eq. 19) and the value of V into Eq. 14 gives


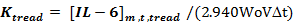


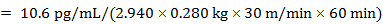


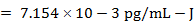
……………………………………........……....20

This indicates that a high quality exercise will trigger the release of myokine directly proportional to the work done by the subject. The “strenuosity coefficient” or “the myokine releasing coefficient” is 7.154×10^-3^ pg/mL-J.

In contrast, the data obtained from the swimming exercise were: [IL-6]_n_ = 6.57 ng/mL for the normal sedentary; IL-6 for DRCKD sedentary = 9.80 pg/mL; for 30 min exercise control = 4.78 pg/mL; and for DRCKD+ 30 min exercise = 7.37 pg/mL. For the 60 min exercise the corresponding values were 4.83 and 7.50 pg/mL, respectively. Thus the residual myokine for the 30 and 60 min exercise are -1.79 pg/mL (4.78-6.57 pg/mL) and -1.74 pg/mL (4.83-6.57 pg/mL). Fig. 6 indicates no myokine release by the 30 min- or 60 min- swimming exercise, and no any difference was found between the 30 min and the 60 min swimming trainings, either for the swimming controls or the DRCKD + swimming exercise. However the IL-6 consumption rate can be calculated. For the sedentary and the 30 min swimming control, we have


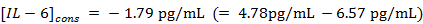
.......................21

Or from the DRCKD data


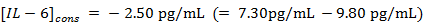
........................22

Correspondingly, the consumption rates are 0.0597 pg/mL-min or 0.0833 pg/mL-min.

Looking back to Eq. 3, the release term can be neglected to give


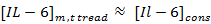
…………………....……...........…....................23

Thus assuming V = 2 m/min, ∆t = 30 min, we have


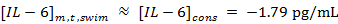
...........................................24


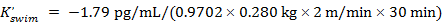

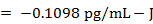
………………………………….......………………....25

Implicating swimming exercise does not release myokine, instead it directly consumes plasma IL-6 in proportional to the work expenditure. The consumption coefficient K’_swim_ is -0.1098 pg/mL-J. Here the symbol K’_swim_ was used instead of the “strenuosity coefficient” or “the myokine releasing coefficient” K_swim_.
